# Host Immunity and Inflammation to Pulmonary Helminth Infections

**DOI:** 10.3389/fimmu.2020.594520

**Published:** 2020-10-20

**Authors:** Jill E. Weatherhead, Pedro Gazzinelli-Guimaraes, John M. Knight, Ricardo Fujiwara, Peter J. Hotez, Maria Elena Bottazzi, David B. Corry

**Affiliations:** ^1^ Department of Medicine, Infectious Diseases, Baylor College of Medicine, Houston, TX, United States; ^2^ Department of Pediatrics, Pediatric Tropical Medicine, Baylor College of Medicine, Houston, TX, United States; ^3^ National School of Tropical Medicine, Baylor College of Medicine, Houston, TX, United States; ^4^ Laboratory of Parasitic Diseases, NIAID, National Institutes of Health, Bethesda, MD, United States; ^5^ Department of Medicine, Pathology and Immunology, and the Biology of Inflammation Center, Baylor College of Medicine, Houston, TX, United States; ^6^ Departamento de Parasitologia, Universidade Federal de Minas Gerais, Belo Horizonte, Brazil; ^7^ Texas Children’s Center for Vaccine Development, Houston, TX, United States; ^8^ Department of Molecular Virology and Microbiology, Baylor College of Medicine, Houston, TX, United States; ^9^ Department of Biology, Baylor University, Waco, TX, United States; ^10^ Hagler Institute for Advanced Study at Texas A&M University, College State, TX, United States; ^11^ Department of Medicine, Immunology, Allergy, Rheumatology, Baylor College of Medicine, Houston, TX, United States; ^12^ Michael E. DeBakey VA Center for Translational Research in Inflammatory Diseases, Houston, TX, United States

**Keywords:** hookworm, *Ascaris*, *Schistosoma*, lung pathologies, inflammation, host immune response

## Abstract

Helminths, including nematodes, cestodes and trematodes, are complex parasitic organisms that infect at least one billion people globally living in extreme poverty. Helminthic infections are associated with severe morbidity particularly in young children who often harbor the highest burden of disease. While each helminth species completes a distinct life cycle within the host, several helminths incite significant lung disease. This impact on the lungs occurs either directly from larval migration and host immune activation or indirectly from a systemic inflammatory immune response. The impact of helminths on the pulmonary immune response involves a sophisticated orchestration and activation of the host innate and adaptive immune cells. The consequences of activating pulmonary host immune responses are variable with several helminthic infections leading to severe, pulmonary compromise while others providing immune tolerance and protection against the development of pulmonary diseases. Further delineation of the convoluted interface between helminth infection and the pulmonary host immune responses is critical to the development of novel therapeutics that are critically needed to prevent the significant global morbidity caused by these parasites.

## Introduction

Helminths are multicellular parasitic organisms belonging to a diverse taxonomic group of metazoans that compromise the phylum Platyhelminths, known as flatworms, including cestodes and trematodes, and Nematoda, known as roundworms, including *Ascaris*, hookworm, whipworms, filarial parasites, and others. Helminths overwhelmingly infect people living in extreme poverty in tropical and sub-tropical regions. Helminths cause significant disease burden globally, particularly in young children, infecting 1.5 billion people worldwide, nearly 20% of the world’s population (1). Together, the impact of helminth infections contributes to more than 12 million disability adjusted life years (DALYS), a measure of significant global mortality and morbidity (2). Helminth induced morbidity is largely due to the direct impact of the helminth on host tissues and indirectly from the host inflammatory response reflecting the complex helminth-host interface.

Host organs such as the lungs are a frequent target of helminth infection. For some human helminth infections, such as paragonomiasis, the adult stage of the parasite takes up its final residence in the lung (3). More commonly, the lung hosts the migratory pathways of the helminth larval stages either through the lung parenchyma or vasculature. As a general principle, helminth tissue invasion causes profound mechanical and chemical damage to the human lung and is linked to vigorous host inflammatory responses. The consequences are huge but the global burden of pulmonary disease as a result of helminths remains unknown.

A coordinated type-2 innate and adaptive immune response aimed at pathogen containment and management of tissue restoration occurs during helminth larval migration. This highly regulated type-2 immune response is communicated through the production of the cytokines interleukin (IL)-4, IL-5, IL-9, IL-10, and IL-13 and chemokines targeting the recruitment and activation of immune cells (4). In addition to the direct tissue damage incurred from the worm itself, helminths also release inflammatory mediators, typically through shedding of the outer chitin layer during molting and through release of soluble excretory-secretory (ES) product or extracellular vesicles (EVs) that contain immunomodulatory proteins including proteases and protease inhibitors, glycolytic enzymes, allergens, and lectins (5, 6). ES product and EVs are released by helminths at all stages of development, and can have an impact on the local environment in a paracrine-like mechanism, but also can influence the immunologic milieu in distant tissues (7, 8). The antigen profile of ES product is diverse amongst helminthic species but remains essential for the maturation and migration of these organisms (9–11). Because parasites are macropathogens, unable to be phagocytized by classic antigen presenting cells (APC), antigens within ES product or EVs are a major mode of communication with the host immune system leading in some cases to immune activation and in others to immunmodulation (12). The variation in antigenic components and concentration within ES product and EVs in different helminths and at different developmental stages contributes to the challenges in understanding the direct relationship between helminth derived factors and host immune responses. Given the link between helminth products, helminth survival, and modification of the host immune response, understanding the communication mechanism between the helminths and host may provide targets for future therapeutic interventions and highlights the need for further investigation.

### Helminth Life Cycles

The impact of helminths on the host lungs is varied and largely depends on the burden of disease and the life cycle of the helminth species. Large burden of helminth disease contributes to a greater degree of tissue damage and a more profound immunologic response aimed at larval control and tissue repair (13). Additionally, helminths have distinct, complex life cycles, which support maturation of larvae into adult worms, as they migrate through varying host tissue compartments including the lungs (14). Several helminths including *Ascaris*, *Strongyloides*, and hookworms (*Necator americanus*, *Ancylostoma duodenale*) have mandatory but transient life cycles that involve larval migration through human lung tissue as an essential larval developmental step. In contrast, *Toxocara*, *Dirofilaria*, and *Echinococcus* larvae, all of which are zoonotic infections, travel to the human lungs and are unable to complete their life cycle; while filaria (*Wuchereria bancrofiti* and *Brugia malayi*) and *Schistosoma* larvae travel through the pulmonary vasculature (15, 16). *Anisakis* and *Trichinella* larvae do not typically migrate to the lungs but do induce a profound systemic inflammatory response during their migration cycle that impacts lung function. The purpose of the species-specific life cycle, particularly one that involves the lungs, remains unclear. Despite the extreme energy expenditure endured during migration, larval migration likely provides a survival advantage allowing for more rapid maturation and larger body size for some species and may also serve as an immune-evasion mechanism during larval development (13, 17). However, the transient migration process of helminths can have a significant impact on the host lungs through activation of the pulmonary and systemic immune responses.

### Helminth Associated-Lung Diseases

**Figure 1:**
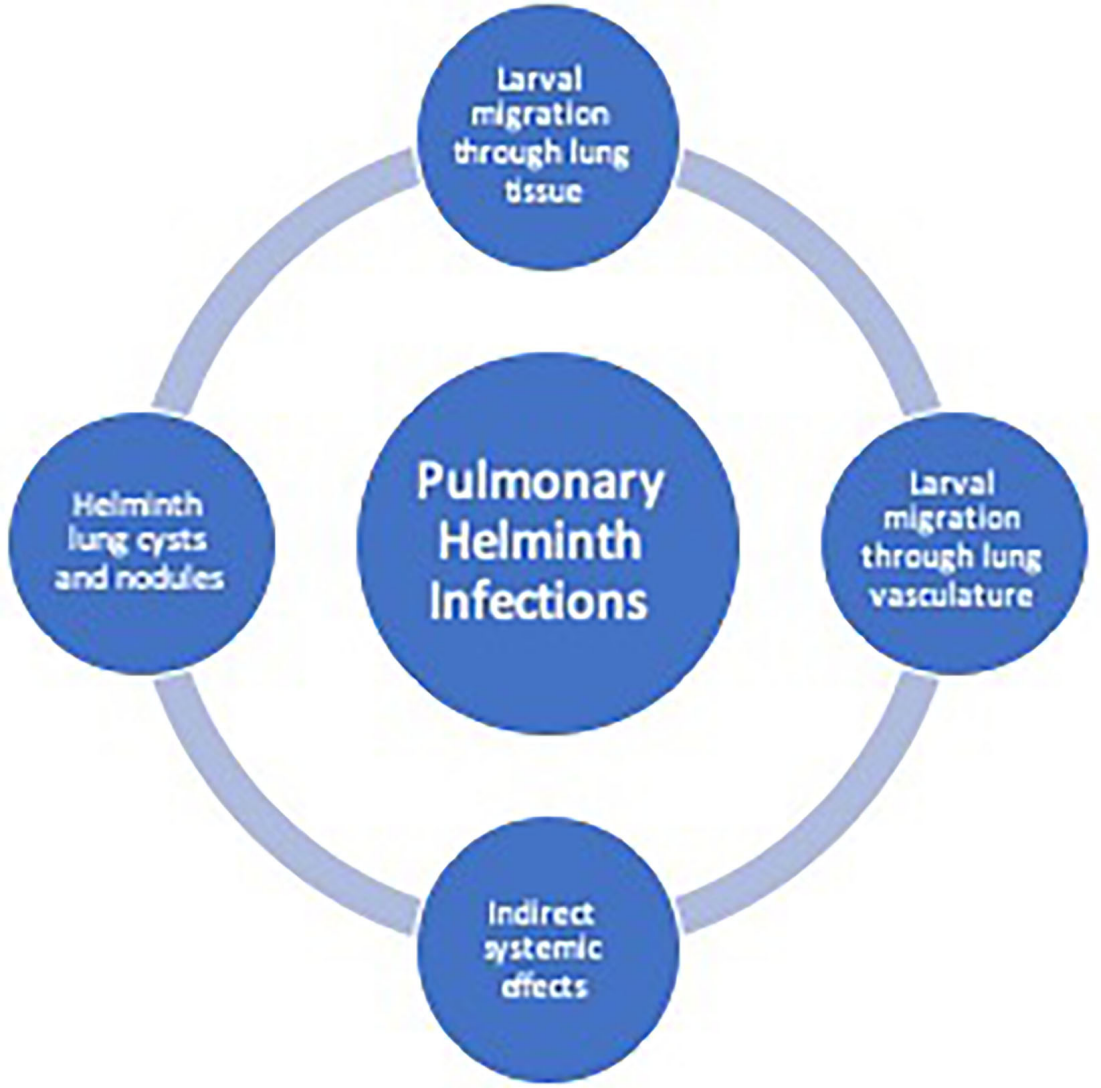
Helminth-induced pathogenesis of human pulmonary disease. Helminth infections can cause pulmonary pathology due to larval migration through the lung vasculature or through the lung tissue causing a diffuse lung disease. Additionally, trapped larvae or eggs can cause focal lesions within the lungs. Indirect systemic effects from helminths may cause pulmonary disease from release of parasite-derived or host-derived factors leading to system inflammation.

Lung pathology induced by helminths occurs in three general categories: 1) diffuse lung disease, 2) focal lung lesions 3) and systemic inflammatory responses and hypersensitivity (**Table 1**). Helminths that cause lung disease typically have either a larval migratory phase directly through the lung parenchyma or through the lung vasculature, form cyst, and nodules in the lung tissue or have indirect systemic effects on the lungs (**Figure 1**).

**Table 1:** Lung pathology induced by helminth infection.

Lung Pathology	Organisms	Lung Appearance	Symptoms
Diffuse Lung lesions	*Schistosoma* spp. *Ascaris lumbricoides* *Ascaris suum * *Wuchereria bancrofti* *Brugia malayi* *Strongyloides stercoralis* *Necator americanus*, *Ancylostoma duodenale* *Toxocara canis* and *cati*	Transient Diffuse lung infiltrates eosinophilic pneumonia	Coughing and wheezing Respiratory failure
Focal Lung lesions	*Dirofilaria* *Paragonimus* *Echinococcus spp* *Schistosoma* spp.	Cysts Single lesions	Asymptomatic Hemoptysis Cough
Systemic Inflammatory Response	*Anisakis simplex* *Trichinella spiralis*	none	Anaphylaxis Dry cough Dyspnea

#### Diffuse Lung Disease

Organisms that have a larval migration stage through the lung parenchyma include the major soil-transmitted helminths, *Ascaris lumbricoides* and *Ascaris suum*, *Strongyloides stercoralis*, hookworm (*Necator americanus*, *Ancylostoma duodenale*), and the zoonotic infection *Toxocara canis* and *cati *(14). These interactions can lead to transient, diffuse lung infiltrates (14), and eosinophilic pneumonia termed Löeffler’s Syndrome (18, 19). Similar to allergic diseases such as asthma, this syndrome is characterized by a severe type-2 immune response with eosinophilia, goblet cell hyperplasia, increased mucus production and manifests clinically as coughing and wheezing and, in severe cases, respiratory failure (13). Given the similar clinical features, helminths such as ascarids may be a major cause of allergic airway disease globally (20). In addition, there are clinical consequences due to mechanical and chemical tissue destruction from these pulmonary helminth invasions, which can produce manifestations that resemble chronic obstructive pulmonary disease (COPD) (21).

Moreover, pulmonary vascular helminths, including filaria (*Wuchereria bancrofti* or *Brugia malayi*) and schistosomes also induce diffuse lung disease. The filarial parasites, for instance, cause a severe pulmonary syndrome called Tropical Pulmonary Eosinophilia (TPE) as a result of marked immunologic hyperresponsiveness to microfilariae trapped in the pulmonary vasculature. Within the pulmonary vasculature, microfilariae degenerate and release antigenic components eliciting a robust local inflammatory processes (22). Clinical manifestations include nocturnal cough, dyspnea, and wheezing in addition to interstitial infiltrates on chest radiograph, predominantly restrictive but also obstructive lung function abnormalities, peripheral blood eosinophilia, and markedly elevated levels of anti-filarial antibodies (23, 24). Other examples of vascular helminths that may trigger diffuse lung diseases are *Schistosoma* spp., primarily associated with *Schistosoma mansoni*, which can lead to different pathways of disease from either the larvae or the eggs. The larval migratory stage known as lung-stage schistosomula sometimes leads to an acute febrile illness known as Katayama fever which can include fever, rash, cough and associated diffuse, interstitial pulmonary infiltrates secondary to release of larvae derived products (25, 26). Additionally, chronic pulmonary schistosomiasis can cause nodular lesions composed of granulomatous inflammation from egg deposition in lung tissue appearing as “ground-glass” on pulmonary imaging (27). Late stage, chronic schistosomiasis can lead to pulmonary arterial hypertension (Sch-PAH). The etiology of Sch-PAH is unknown but may be due to egg deposition in pulmonary vessels causing vascular inflammation or due to portal hypertension. PAH most commonly presents clinically as dyspnea and reduced exercise tolerance. Given the widespread distribution, schistosomiasis may be a leading cause of PAH globally (27–29).

#### Focal Lung Lesions and Cysts

Other organisms such as *Dirofilaria* and *Echinococcus*, in which humans are accidental hosts and do not permit completion of the life cycle, worms are sequestered in the lungs and induce an inflammatory response manifesting as focal lung lesions (13). *Dirofilaria immitis*, the dog heartworm, cause formation of granulomas in the pulmonary vasculature seen on pulmonary imaging as “coin lesions”. Likewise, Echinococcus causes fluid-filled hydatid cysts in the lungs (13). *Paragonimus* also forms focal lung lesions. Larvae migrate to the lungs and develop into adult worms in pulmonary cysts manifesting clinically as cough, fever, chest pain and hemoptysis (16). Because paragonimiasis geographically overlaps with tuberculosis in East Asia, it is not uncommon to confuse these two causes of hemoptysis (30).

#### Indirect Effects of Systemic Inflammation

Several helminths, which do not directly infect host lungs, cause significant systemic inflammation through parasite-derived immunomodulatory molecules (13). Heavy infection with *Anisakis simplex*, thorough ingestion of raw or uncooked fish or handling fresh fish, can cause allergic airway disease or life-threatening anaphylaxis despite no direct contact with the host lungs. Fish processing workers specifically are at increased risk of developing *Anisakis*-induced bronchial hyperresponsiveness from consuming, touching or even inhaling *Anisakis* proteins (31). *Trichinella spiralis* larvae obtained from consuming undercooked pork or wild game can migrate through the lung vasculature on the way to striated muscle causing a dry cough (32). However, more commonly lung pathology occurs from either the systemic release of larval products during acute trichinellosis causing cough and dyspnea or from larvae developing into cysts in the chest wall accessory muscles and the diaphragm impacting lung dynamics (33).

These distinct clinical manifestations occur regularly in helminth endemic areas or can occur seasonally based on region specific transmission patterns (34). However, understanding the role of the host immune response to helminths and the resulting clinical impact on human lungs can be challenging and has lead to contradictory outcomes in clinical studies (20).

## Helminths and the Host Immune Response

The challenge in gaining an in-depth understanding of the host innate and adaptive pulmonary immune response to helminths is multifactorial. Human studies have led to varying outcomes based on inconsistent diagnostic capabilities, variation in study populations, seasonality of infection, genetic predisposition, population age, and disease burden. As a result, animal models have been used to gain a greater understanding of the host pulmonary immune response to helminths. *Nippostrongylus brasiliensiss, Strongyloides venezuelensis*, *Schistosoma mansoni*, *Brugia malayi*, and *Ascaris suum* have all been used to evaluate the impact of helminths on host lung function in animal models (35, 36). These animal models are critical to elucidate the complex balance between helminth induced innate and adaptive lung immunity. Mouse models of Nippostrongylus, *Schistosoma*, and *Ascaris* have been especially helpful in elucidating immunological mechanisms. It is critical to dissect the complex lung innate and adaptive immune response as a result of helminth infection in order to inform on-going therapeutic and vaccine development that is needed to reduce global morbidity.

### Innate Immune Response in the Lungs to Helminthic Infections

Early recognition of helminths by the host pulmonary innate immune response is critical for disease control. Helminth molecules, glycans and lipids, serve as pathogen-associated molecular patterns (PAMPs). Recognition and processing of helminth PAMPS by APCs through pattern recognition receptors (PRR) such as toll-like receptors (TLR), C-type lectins and intracellular nucleotide-binding oligomerization domain-containing protein (NOD)-like receptors are initial steps in the host innate immune response engagement (17, 37, 38). Intracellular signaling as a result of PRR activation, leads to release of cytokines, particularly IL-4, IL-5, IL-9, IL-13, IL-25, IL-33, and IL-17 as well as chemokines that propagate the host immune response. Cytokines and chemokines activate and recruit additional innate inflammatory cells including neutrophils, macrophages, basophils, eosinophils, dendritic cells, mast cells, natural killer (NK) cells, and type 2 innate lymphoid cells (ILC2) to the lungs, further propagating the innate immune response (13, 39). Helminths also expel cytokine- and chemokine-like natural products and molecules displaying immunologic mimicry, further aiding in cellular recruitment and activation. The recruitment and activation of innate cells in coordination with epithelial cell release of alarmins (e.g., IL-33; HMGB-1) and cellular hyperplasia, goblet cell production and release of intraluminal mucous and smooth muscle contraction are all necessary for the eradication of parasites from host lungs. A variety of specialized innate cells direct type-2 immune polarization and subsequent synchronization with adaptive immune pulmonary responses against helminth infections. The major innate immunity effector mechanisms to helminth infections include the following major elements.

#### Complement System

The classical, lectin and alternative complement pathways are prominently activated in early helminth pulmonary infection (40). Mice deficient in C3 infected with *Strongyloides* have increased larval survival (41). Despite activation of complement during helminth infection, helminths are able to evade complement mediated immunity potentially through binding complement inhibitory protein factor H, during molting, a process allowing for shedding of the larval outer layer (40), and by expressing surface proteinases that continuously degrade complement proteins binding to the helminth cuticle. While not necessarily playing a large role in opsonization and pathogenic killing, complement remains an important regulator of leukocyte-mediated immunity during helminth infection. Specifically, C3a and C5a act as chemotactic factors aiding eosinophil recruitment (40). Despite all three pathways being engaged, the independent role of the three individual pathways may differ depending on the helminth species, helminth developmental stage and the location of infection (40). In *N. brasilliensis* infection, the alternative pathway, mediated by hydrolysis of C3, plays a major role in eosinophil adherence in the infective larval stage (L3) occurring in the host lungs. In contrast, during the adult stage of *N. brasilliensis*, which resides within the gastrointestinal tract, lectin pathway plays a larger role in helminthic control (40).

#### Mucosal Barrier (Epithelium, Smooth Muscle, Mucus Production)

Direct helminth-induced damage to and death of lung epithelial cells can be one of the initial triggering events in helminth infection, leading to release of epithelial derived alarmins IL-25, IL-33, thymic stromal lymphopoietin (TSLP), and chemokines CXCL1, CXCL2, CXCL8, and eotaxins (42, 43). These alarmins signal to innate cell populations such as Innate lymphoid cells type 2 (ILC2), basophils, eosinophils, neutrophils, macrophages, and dendritic cells during the early phases of infection (43). The influx of type-2 cytokines into the lung compartment as a result of innate immune cell activation, particularly through the action of IL-4, IL-9, and IL-13, aids in goblet cell hyperplasia andmucin production needed for helminth expulsion and epithelial turn-over and repair (37, 43). In *Schistosoma mansoni* infection in the lung, rapid influx of IL-9 leads to robust generation of goblet cell hyperplasia (44). IL-4 receptor *alpha* (IL-4Rα) signaling *via* IL-4 and IL-13 cytokines also plays a role in mucin production and smooth muscle responsiveness during *Nippostrongylus* infection (45). Smooth muscle cells responding to IL-4Rα activation on smooth muscle leads to airway hyperresponsiveness, the exaggerated tendency of the airway to constrict, during *Nippostrongylus* infection in addition to T cell recruitment to the lungs (45). Mice deficient in smooth muscle IL-4Rα lack coordination of acetylcholine responsiveness with reduced M3 muscarinic receptor expression, delayed goblet cell hyperplasia, reduced type-2 cytokine production, and have a delayed ability to expel *Nippostrongylus (* 46). Independent of IL-4Rα signaling, secreted proteins Ym1 and Restin-link molecule *alpha* (RELMα/Fizz1) from epithelial cells in the lungs contribute to lung repair through inducing IL-17A and neutrophilic infiltration promoting type-2 immunity and remodeling (47). Pulmonary tuft cells, termed brush cells, along the airway epithlium contain microvilli and potentially function as chemosensory cells playing an additional role in innate epithelial immunity (48). Tuft cells in the intestines have been shown to be involved in the generation of type-2 immunity to helminths (49). However, more research is needed to evaluate the interaction between helminths and pulmonary tuft cells.

#### Neurons and Neurotransmitter Signaling

The role of the neuroimmune network in helminth infection has more recently been highlighted as a critical modulator in helminth control and tissue repair particularly at heavily innervated mucosal sites (50). Pulmonary neuroendocrine cells, located at airway branching points release neuropeptides and neurotransmitters that influence smooth muscle tone (51). Bovine lungworm infection, *Dictyocaulus vivparus*, leads to upregulation of nictotinic acetylcholine receptors and increased cholinergic signaling on immune cells including epithelium, leukocytes, and macrophages (52). Additionally, helminth-derived acetylcholinesterase (AChE) has been identified in ES product of lung stage helminth larvae in different species. AChE secreted by *Nippostrongylus* regulates hydrolysis of endogenous Ach in mucosal tissue and co-localizes with cholinergic mucosal neurons that express the neuropeptide neuromedin U (NMU) suggesting that helminth derived products are involved in neurotransmitter modulation in mucosal tissue (50, 52, 53). NMU is particularly involved in coordination of the host innate response in the lungs during helminth infections. Neuron stimulation by either host alarmin IL-33 or IL-25 or *Nippostrongylus* ES product leads to direct neuronal release of NMU showing that neurons are sensing host damage and larval products (54). High concentrations of IL-25 in combination with NMU are associated with enhanced expression of type-2 cytokines IL-5 and IL-33 (55). Furthermore, intranasal administration of NMU has been linked to initiation of type-2 immune responses such as increased eosinophils, decreased tissue hemorrhage, enhanced mucus production and reduced infectious burden (50, 54). This may in part be an indirect effect of NMU given its role in enhancing maturation, proliferation and cytokine expression of lung ILC2s. ILC2 and NMU seem to have an inter-dependent relationship during helminth infection in the lungs. ILC2 selectively express neuromedin U receptor 1 (*Nmur1*), deletion of which impairs type-2 immune response and parasite control (54). NMU neuroimmune effects during helminth infection is counter regulated by the neuropeptide CGRP. The cognate receptor of CGRP is highly expressed on IL-5 secreting ILC2 populations, and when activated, decreases ILC2 populations (56).

#### ILC2s

ILC2s are innate tissue-resident cells particularly abundant at mucosal barriers including the lungs. They are found in high concentrations around the adventitia of lung bronchi and large vessels and co-localize with subsets of dendritic cells, T regulatory (Treg) cells, adventitial stromal cells, and mesenchymal fibroblasts (57). ILC2 activation and proliferation in the lungs is dependent on the release of alarmins IL-25, IL-33, and TSLP, in addition to the neuropeptide NMU. ILC2s also express IL-4Rα enabling the cells to respond to the local type-2 cytokine milieu during helminth infection. Responding to IL-4 and IL-13 signals leads to further cellular expansion of ILC2s and a robust release of type-2 cytokines particularly IL-5 and IL-13 in a STAT6-independent mechanism (58, 59). The significant influx of IL-5 and IL-13 supports eosinophil recruitment, epithelial cell hyperplasia, and goblet cell hyperplasia (60). STAT6 is also not required for IL-13 production in ILC2s response to *Nippostrongylus* in the lungs (59). Once thought to be a homogenous population, single cell analysis during *Nippostrongylus* infection demonstrates significant heterogeneity within ILC2 populations in the lungs (56). Transient IL-25 responsive ILC2 (iILC2) are circulating ILC2s that mobilize to lung tissue in response to chemokines and cytokines (58, 61). Basic Leucine Zipper ATF-Like Transcription Factor (BATF), an AP-1 superfamily transcription factor, is critical for activation of iILC2 during *Nippostrongylus* infection (58). BATF-deficient *Nippostrongylus* infected mice lack iILC2 in lungs, have reduced early influx of type-2 cytokines and impaired mucosal barrier integrity. Conversely, tissue resident ILC2 (nILC2) are IL-33 responsive and not BATF- dependent (58, 61). Migration of ILC2 to the lungs may be secondary to increased expression of prostaglandin D2 (PGD2) receptor CRTH2 (chemoattractant receptor-homologous molecule) on the cell service (62). IL-33 coordinates the PGD2–CRTH2 pathway further regulating ILC2 migration patterns to the lungs (63).

#### Granulocytes (Basophils, Mast Cells, Eosinophils)

In addition to ILC2, basophils, mast cells, and eosinophilia constitute IL-4– and IL-13–producing cells of the innate immune system during lung helminth infection. The role of basophil and eosinophil production of IL-4 and IL-13 is sufficient enough to induce allergic airway disease in defense against migrating worms (64).

Basophils are granulocytes that produce type-2 cytokines including IL-4, IL-5, and IL-13 in addition to histamine, leukotrienes, and prostaglandins during degranulation as a result of helminth infection (65). Production of IL-4 by basophils is STAT-6 independent. *Nippostrongylus* infection as well as *Schistosoma* egg antigen (SEA) increase the number of basophils in the lungs as a result of FcR cross-linkage by IgE or IgG, complement activation of C5a or cytokine stimulation through T cell derived IL-3 or IL-18 (66–69). As a result, basophils can potentially serve as an early source of IL-4 during helminth lung infection prior to T cell activation. *Schistosoma* ES product IPSE/alpha-1 can trigger basophil production of IL-4 and IL-13 and influence cellular commitment toward type-2 immunity such as alternatively activated macrophage (AAM) formation, smooth muscle activity, goblet cell hyperplasia, and eosinophilic infiltration into the lungs (65, 70). Basophils are also capable of processing antigen to naïve CD4 T cells and promoting Th2 differentiation *via* MHC Class II expression (65, 71). While basophils are not essential for Th2 differentiation they are likely a critical initial trigger in the type-2 immune cascade (65). Recently, up-regulation of Notch2 receptor in basophils was shown to cause increase cytokine production, including IL-4 and IL-6, in intestinal helminth infections. Moreover, basophil-intrinsic Notch signaling promoted worm clearance and type 2 inflammation in the cecum (72). Whether basophil Notch signaling occurs in the lungs remains unclear.

Mast cells located along the epithelial lining of the host lungs additionally play a role in helminth infection. At baseline, mast cells are rare resident cells in host lung tissue but can rise significantly during helminth infection. However, the inciting chemoattractants that induce mastocystosis in the lungs during helminth infection remains largely unknown (73). IL-9 signaling through IL-9R on mast cells is an important factor for maturation and activation of mast cells. Depletion of IL-9 during *Schistosoma mansoni* infection demonstrates 8-fold fewer mast cells in the lungs (44). Additionally IL-3, derived from varying cell sources including T cells, may contribute to further influx of mast cells. In IL-3 deficient, *Strongyloides* infected mice, mast-cell development is stalled and parasite killing is impaired (67). Degranulation and rapid release of inflammatory mediators (histamine, leukotrienes, and prostaglandins), cytokines (IL-4, IL-6, and TNFα), and proteases (mcpt1 and mcpt2) occurs secondary to high affinity mast cell FcϵR1 bound to IgE, which plays an important role in helminth killing (11, 43). However, release of these mediators including IL-4 from mast cells is not required to generate a robust type-2 immune response. Mast-cell deficient mice infected with helminths can still generate normal type-2 immune response in the lungs (65).

Eosinophils are critical effector cells during helminth infection through direct killing of parasites and, potentially acting as APCs directing T cell differentiation to type-2 immune cells, although this function remains controversial (39, 74, 75). The early presence of helminth larvae in the lungs triggers rapid eosinophilic activation and recruitment through the release of chemoattractants eotaxins and MIP-1a as well as cytokine IL-5 production (76). Eosinophilic degranulation occurs with engagement of the FcϵR1 on eosinophils surface to Fc of IgE bound parasites (11). Eosinophilic cellular cytotoxicity of helminthic larvae in the lungs occurs through release of major basic protein (MBP) and eosinophil peroxidase (EPO)-dependent mechanisms particularly during secondary infection and through induction of histamine release from mast cells (75, 77). The release of eosinophil DNA-based extracellular traps (EETs) may be an additional mechanism of helminth larvae-killing or immune response against extracellular pathogens (78). The eosinophilic immune response restricts larval development and reduces parasite burden in the lungs particularly in secondary exposure to helminths or in hosts with allergen presensitization (79). Presensitized lung tissue and airways in HDM-allergic lungs or after multiple helminth exposures, contain IL-4 and IL-13-rich AAMs and eosinophil-dominated type 2 cellular infiltration, which dramatically reduces the burden of disease, including lower hemorrhage and mechanical damage in the tissue as a result of parasite migration through tissue (76, 79). Likewise, deletion of eosinophils in animal models has been shown to increase parasite survival (38, 39). Reduction of eosinophils through anti-IL-5 or anti-CCR3 monoclonal antibody blocks innate protective immunity to *Strongyloides* and leads to reduced control of larvae (39). However, eosinophils may not be essential for larval killing as neutrophils provide redundency (77). In the complete absence of eosinophils during *Strongyloides* infection, neutrophils were capable of partial control of infection (39). While eosinophils release IL-4 and aid in type 2 T cell polarization, eosinophil deficient mice still mount a normal T helper type 2 (Th2) response to helminth infection (65).

#### Neutrophils

Neutrophils also perform as effector cells during helminthic infection in the lungs. Facilitated by the release of myeloperoxidase, neutrophils can independently kill *Strongyloides* larvae in the lungs (39). Interestingly, *Strongyloides* larvae alone can directly induce the activation and recruitment of neutrophils to infected tissues (80). *Strongyloides*-specific molecules released during the larval stage promote neutrophil release of chemokines MIP-2 and KC, further enhancing recruitment of neutrophils (39). Additionally, chitinase-like proteins Ym1 and Ym2 lead to expansion of IL-17A–producing γδT cells with increased IL-17A production and subsequent neutrophil recruitment. The enhanced neutrophil recruitment prevents parasite survival but at the expense of enhanced lung injury (81). Likewise *Anisaki* release of ES products elicits neutrophil recruitment to the lungs *via* production of IL-6, IL-8, and CXCL1 which has been shown to further enhance lung inflammation and contribute to lung disease during anisakiasis despite no lung larval migratory phase (82). Such tissue damage is not seen to the same degree with type 2 immunity-dependent, eosinophil predominant inflammation, suggesting that type 2 immunity may have evolved to provide control over chronic, inescapable parasitic infections while minimizing bystander tissue injury.

#### Natural Killer Cells

NK cells contribute to helminthic control in the lungs through direct cytotoxicity and cytokine production. NK cells expand early in helminth lung infection potentially secondary to recognition of helminth ES product (83, 84). Filarial infective-stage larvae and microfilariae modulate NK cell activation and release of cytokines including gamma interferon (IFN-γ) and tumor necrosis factor-α (TNF-α) (85). In filarial disease animal models, *in vivo* depletion of NK cells increased worm burden in the pleural cavity and influenced IL-4 and IL-5 plasma levels (84). Likewise, pulmonary schistosomiasis increases NK cells within the lungs and NK cells localize near *Schistosoma* pulmonary granulomas (86). NK cells additionally promote dendritic cell maturation through IL-12 production and directly interact with T cells providing a link between innate and adaptive immunity (86).

#### Professional APCs (Macrophages and Dendritic Cells)

Dendritic cells (DCs) serve as mediators in the innate and adaptive orchestration of type-2 immunity during lung helminth infection (87). DCs recognize helminth ES-derived antigens or parasite surface molecules. Proteins and lipids that are highly glycosylated are recognized by different innate PRR including TLR, c-type lectin receptors, and NOD-like receptors (11, 87, 88). DCs capture helminthic antigen and present to T cells propagating the host type-2 immune response (11). *Schistosoma* and filaria both activate TLR4 on dendritic cells to engage type-2 immunity in the lungs potentially through reduction in dendritic cells ability to produce IL-12. However, evaluation of th immunologic pathways involving TLR4’s role in the development of type-2 immunity is on-going (11).

Activated macrophages in the lungs serve as APCs that aid in transitioning to a type-2 adaptive immune response during helminthic infection and selectively polarize toward type-2 mediated AAMs. In the context of a predominant type-2 immune response environment, IL-4 and IL-13 signaling *via* the STAT6 pathway induce expression of mediators that promote AAM. The IL-4/IL-13 and STAT6 signaling pathway allow for upregulation of genes associated with AAM (YM1, YM2, RELMα, ARG1) and upregulation of class II MHC on macrophages to further promote type-2 immune responses (89, 90). Amplified concentrations of AAM during helminth infection in the lungs impedes parasite migration and are sufficient to kill *Nippostrongylus* *in vitro (* 80). Additionally, AAM associated RELMα specifically supports tissue repair, extracellular matrix turnover and homeostasis and is essential to prevent fatal lung damage as a result of helminth infection in the lungs (89, 90).

**Figure 2:**
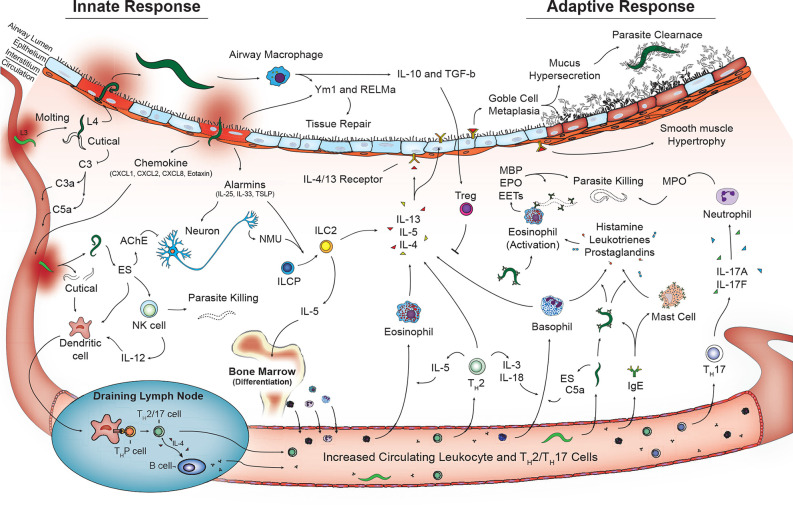
Activated innate and adaptive immune pathways in the lungs during helminth infection. Innate phase of immune activation to helminths in the lungs is mediated by antigen presenting cells including dendritic cells and macrophages in addition to release of alarmins from damaged epithelium that drive a type-2 immune response. Release of type-2 cytokines IL-4, IL-5, and IL-13 contributed from granulocytes and type 2 innate lymphocytes promote a lung phenotype with goblet cell metaplasia and mucus hypersecretion and smooth muscle hypertrophy. T lymphocytes including Th2, Th17 in combination with B lymphocyte aiding immunoglobulin class switching further contribute to type-2 immune response promoting parasite killing and clearance as well as tissue repair and recovery.

Additional cells that play a role in innate immunity including γδT cells (81) and coagulation factors (platelets, thrombin, fibrinogen) (91, 92) may also contribute to helminth disease control and tissue damage in the lungs. However, additional studies are required. Overall, the coordinated work of innate cells play a critical role in early recognition and termination of helminth infections as well as initiation of tissue repair in the lungs. However, perhaps the most important role of the innate cells during pulmonary helminthic infections is engagement and activation of the adaptive immune response (**Figure 2**).

### Adaptive Immune Response in the Lungs to Helminthic Infections

Innate immunity is not just important as the first line of defense but also influences the nature of the adaptive response (93), by creating environments with signature cytokines that have the ability to drive the proliferation and differentiation of the effector T helper (Th) cells. In response to helminth infection, naive CD4^+^ T‐cells differentiate into several possible effector subsets, including Th2, Th17, T regulatory cells (Treg), and T follicular helper cells (Tfh) as well as influence the maturation of B cells and isotype switching of immunoglobulins favoring a type-2 immune response (94). Conversely, Th1 cells that secrete principally IFN-γ are activated most commonly in intracellular parasitic infections such as protozoa (e.g., *Leishmania* and *Plasmodium*) and are not predominately linked to helminth infections with some exceptions (95). Several cytokines, mostly originated from innate cells, and transcription factors tune the differentiation and expansion of these cell populations. Overall, helminth-induced immunoregulation occurs through the induction T cell subsets and B cell maturation influencing the immunologic landscape during helminth infection, providing effector function, tissue repair, and memory in the lungs. Of note, very few studies have analyzed CD8^+^ T cell responses to helminth infection in the lung tissue. While, CD4^+^ T cells classically play a major effector role in pulmonary helminthic disease, CD8^+^ cells can enhance granulomatous inflammation in the lungs to schistosomiasis (96). More detailed analysis of the role of CD8^+^ cells during pulmonary helminth infection are needed. While helminths classically create a type-2 immune response, the specific balance of adaptive immune cells is intrinsically associated with the helminth species, the stage of infection (acuteness or chronicity) and what organ/tissue has been affected. In the lung tissue, as most of the pulmonary helminthic infections are transient, the role of the adaptive response can be divided in 2 phases: helminth control and tissue repair.

#### Helminth Control in the Lungs

During parasitic invasion of the lungs, Th2 effector cells are essential to rapidly polarize and amplify a multifactorial type-2 inflammatory response to control parasite burdens. Although the mechanisms are not fully elucidated, the circumstances that promote the acute Th2 cell protective immunity in the lungs occurs by cross-talk with innate immunity through cytokines, chemokines and growth factors that result in the establishment of a robust type-2 inflammatory environment rich in AAM and dominated by eosinophils. Initial Th2-cell differentiation largely involves the cytokines IL-2, IL-4, and collectively IL-25, TSLP, and IL-33 as well as the transcription factor GATA3 directing effector function in the lungs (95, 97, 98). The Th2 effector response to helminth infections in the lungs is thus typified by the differentiation of antigen-specific CD4^+^ T cells to Th2, high levels of IL-4, IL-5, and IL-13, accompanied by eosinophilia, mucus production, and abundant IgE production, phenotypically similar to allergic airway disease (99).

IL-4–, IL-13–, and IL-5–producing CD4^+^ Th2 cells have been implicated as one of the most important effector mechanism in adaptive pulmonary immunity against helminths. IL-5–producing Th2 cells provide support for IL-5–mediated eosinophil-dependent larval killing. IL-5 enhances the differentiation, maturation, and survival of eosinophils derived from bone marrow precursors (100, 101). In animal models of *Strongyloides stercoralis*, Th2 production of IL-5 is not only required to elicit additional effector function but also to induce protective antibodies (102). IL-4– and IL-13–producing Th2 cells induce STAT6-dependent changes in epithelial-cell function resulting in goblet cell hyperplasia and increased mucus production *via* IL-4Rα and IL-13Rα1 signaling. Additionally, IL-4Rα expression on lung-resident CD4^+^ T cells is required to generate a protective recall immunity to *N. brasiliensis* re-infection (103). In subsequent infection, the presences of primed memory Th2 cells aid in the accumulation of eosinophils expressing high levels of MBP. The recruitment of eosinophils by memory Th2 cells facilitates more rapid reduction of helminth burden in the lungs compared to primary infection (104).

Th2 effector cells and Tfh cells in germinal centers coordinate the humoral response in type-2 inflammation by promoting a helminth-specific B cell response and stimulating B cell class switching to high-affinity IgE, IgG1, and IgG4 (38). Tfh is reliant on Notch signaling in an IL-4 dependent pathway and the transcription factor BCL6. However, deficiency of Tfh does no impact worm clearance, overall IL-4 production or Th2 mediated immunity (105, 106). Antibodies are the quintessential adaptive immune effector molecules and are a central feature of Th2 immunity (97). Moreover, B cell activation has been investigated over the years as the target for effective vaccine development against helminth parasites and antigen-specific IgG antibodies generate a protective adaptive immune response against *Ascaris* parasites specifically (107).

#### Lung Tissue Repair and Restoration

After parasites have migrated through the lungs, Th2 effector cells are critical to activating efficient tissue repair responses. IL-4– and IL-13–producing Th2 cells are important for differentiation and maintenance of AAM in the tissue, encoding genes such as YM1, RELMα, and Arg1, key players in tissue repair and remodeling (108, 109). The crosstalk between Th2 effectors cells and macrophages has been demonstrated in experimental models of the filarial nematode *Brugia malayi.* Filarial infection in recombinase activating gene (RAG) or MHC class II-deficient mice (mice lacking T cells) demonstrate a striking lack of AAM, suggesting that Th2 cells are required for maintenance and full activation of AAM directed repair immunity to helminth parasites (110).

Treg populations, “natural” Tregs and “induced” Tregs, are activated in an effort to dampen host immune response through production of IL-10 and TGF-β during helminthic infection (111). The role of Treg varies depending on the developmental stage and the parasite species. Lung damage, blood clots, damage-associated molecular patterns (DAMPS) as a result of helminth infection in the lungs, promotes differentiation of macrophages and release of macrophage-derived growth factors and anti-inflammatory IL-10 and TGF-β that activate Treg (38). Depletion of Treg heightens Th2 cell proliferation and clearance of infection; however, this is associated with increased lung damage due to prolonged effector activation (112). Conversely, Treg production early in infection can promote worm survival by dampening the acute Th2 effector cell response but promoting tissue integrity (95). In helminth re-infection models, Tregs suppress memory Th2 cells in order to reduce the recruitment of eosinophils into the lungs and prevent excessive lung damage and further pathology (104, 111). Additionally, Treg activation during helminth infection can generate non-specific host immune suppression across tissue compartments and thus can be important in parasitic co-infection models particularly regarding progression of malaria and tuberculosis (112).

Additional T helper subtypes can play important roles during helminthic infection. Driven largely by pro-inflammatory cytokines, IL-6 and IL-17A and the transcription factor RORγt and maintained by IL-23, Th17 cells at mucosal barrier sites aid in mucosal defense and tissue restoration (95). Lung tissue expression of receptors for the Th17 cytokines IL-17A, IL-21, GM-CSF, and IL-22 allows for both protective and pathogenic responses in the lungs to helminth challenge (113). During *Nippostrongylus* infection in the lungs, Th17 activation is associated with early release of IL-17A which aids in IFNγ suppression and type-2 immunity, recruitment of neutrophils and subsequent pulmonary damage and hemorrhage (38, 114). Prolonged Th17 activation and excessive early IL-17A can be associated with immune-mediated disease in the lungs including asthma (114). It is also linked with the extravasation of eosinophils from the bone marrow and eosinophilic infiltrations in host lung tissues. However, once established, IL-17A can also act as a negative regulator of the type-2 response in the lungs (114). On that basis Th17 responses, mediated through IL-17A, may be as important as Th2 responses in the pathogenesis of helminth-inducted pulmonary disease (115). However, Th17-driven IL-22 aids in epithelial cell repair and regeneration of mucosal barrier after helminth infection in the lungs (116). The IL-17/IL-22 axis is critical to the generation of epithelial homeostasis post-infection. IL-6 promotes differentiation of Th22 cells and release of IL-22, which also likely aids in tissue repair although the role of IL-22 in pulmonary helminth infections remains unknown. Additionally, Th9 in gastrointestinal helminth infection has been shown to cause rapid worm expulsion and basophil activation however further investigation is needed in the lungs (117).

#### Systemic Impact of the Adaptive Immune Response

The complexity of the adaptive immune response to helminths is varied and can be influential in co-infection and co-morbid models of disease. Helminth co-infection models with different infected host compartments impacts not only the local adaptive immune response but also the host immune response to helminth infection in distant tissues. *H. polygyrus* intestinal infection induces a IL-33–mediated activation of IL-5 secreting Th2 cells and causes upregulation of IL-5–mediated eosinophils, leading to a significant increase in immune-mediated killing of *N. brasiliensis* larvae in the lungs of the co-infected mice (118). Additionally, co-morbidities may also influence the adaptive immune response to helminth infections. In an animal model of house dust mite (HDM) allergic sensitization, subsequent *Ascaris* infection is mitigated by a primed memory Th2 response in the lungs. Allergen-driven inflammation, increases IL-5– and IL-13–producing Th2 cells in the lungs leading to an IL-4– and IL-13–rich environment that drives the differentiation of lung macrophages toward an AAM phenotype expressing arginase-1, as well as, an eosinophil infiltration. This strict type-2 immune milieu leads to a marked reduction in the number of lung-stage *Ascaris* larvae, reducing the intensity of infection, inducing lower pulmonary hemorrhage and mechanical damage in the lung tissue during larval migration (79). These results suggest a sophisticated and efficient feedback loop among Th2 cells, eosinophils and AAM in coordinating innate and adaptive immunity against lung tissue helminths (95).

#### Implications for Vaccines

Several human helminth vaccines are in phase 1 or phase 2 stages of clinical development, including hookworm infection and schistosomiasis (119). For human hookworm infection, vaccine immunity appears to operate *via* directing anti-enzyme antibody responses to parasite gut digestive enzymes, including a glutathione S-transferase and a hemoglobinase, while schistosome vaccines focus on larval and adult surface antigens (120). Extensive studies in mice show how vaccine immunity against schistosomes and possibly other helminths operate through adaptive immune responses in the lungs. In some cases, these responses actually trap larval helminths in the lung and block further migration (121). Furthermore, *Ascaris* vaccines targeting larval-stage immunodominant proteins that are highly conserved between helminths suggest the possibility of developing a pan-helminth vaccine that may prevent larval migration (122). Therefore, helminth vaccine development targeting effector immunity in the lung may prevent larvae from completing their life cycle while preventing host lung damage.

## Helminths and Immune Modulation

Helminths can directly cause lung pathology as a result of direct larval migration or indirectly through systemic activation of a type-2 immune response. Many of the molecules released from helminths have been associated with development of lung disease, including allergic airway disease. *Ascaris* ABA-1 fatty acid binding protein, a glutathione transferase (GST) involved in distribution of lipids, is released at different stages of *Ascaris* development including the lung stage. ABA-1 has significant homology to mite and cockroach GST and has been linked to the development of asthma (123). Conversely, *Anisakis*, which is the most allergenic helminth and does not typically have a lung larval stage, contains the allergens molecules Ani s 1, Ani s 4, and Ani s 9, in ES product, all of which are proteins central to the development of systemic anaphylaxis and lung pathology (123). Similar proteins are found in the ES product of hookworm and *Schistosoma* spp. that have been linked to lung disease (123). This acute lung pathology can lead to chronic lung abnormalities. *Nippostrongylus* larval migration causes destruction of alveoli, long-term airway hyperresponsiveness and chronic low level hemorrhaging in the lungs through chronically activated AAM and release of matrix metalloproteinase 12 (MMP-12; macrophage metalloelastase) (21). The release of high levels of IL-13 causes persistent type-2 immune polarization of macrophages leading to on-going, chronic lung damage over months (14). However, these acute and chronic changes in the lungs are likely developmental stage- and helminth-specific.

While some larval stages induce end organ disease within the lungs, others, particularly those without larval migration through the lungs or once the larvae develop into adult worms, induce immunomodulation and may prevent underlying inflammatory disease states. Components of ES product as well as secreted EVs can be associated with immune evasion and immunomodulatory mechanisms (5). Helminths can produce mediators within the ES product that influence regulatory immunity using immune mimicry through production of TGFβ cyotkine-like molecules and altering the gut microbiome to promote Treg differentiation (38, 124). Additionally, helminth derived chemokine-like molecules such as IPSE/alpha-1 have also been linked to immune modulation (70). *Ascaris* ES product at larval and adult stages contains protein-1 of *Ascaris suum* (PAS-1) which contributes to increased concentrations of IL-10, a cytokine that is both made by and influences the generation of Treg and thus an immunomodulatory environment (125). Likewise, anti-inflammatory protein-2 (AIP-2) secreted from hookworms, has also been noted to enhance Treg and suppress airway inflammation. This influence over the host immune response balance can lead to long-term protection of lung structure and function (14). Animal models of *Heligmosomoides polygyrus*, a non-pathogenic helminthic infection in the gastrointestinal tract, has been shown to attenuate airway inflammation (38, 126, 127). Helminth-derived immunomodulatory molecules can influence all facets of the host immune response including cytokine and chemokine signaling and gene expression (126). This immunomodulation and immune-mimicry allows parasites to live within the host for extended time periods. Further characterization of helminthic molecules and their cognate host receptors may contribute knowledge in the development of future therapeutic approaches in the treatment of diverse inflammatory conditions.

## Conclusions

Helminth infections remain a significant global issue impacting the lives of billions of people. Several human helminth infections lead to profound morbidity including pulmonary disease. Conversely, some helminths may prevent the development of pulmonary disease. This dichotomy in outcomes in the lungs and the global impact on pulmonary disease is likely helminth-species and helminth-stage specific. Dissecting the intricacies of the parasite-host pulmonary immune response is critical knowledge in order to develop therapeutic strategies to reduce helminth burden worldwide.

## Author Contributions

JW developed, researched, wrote, edited, and reviewed the manuscript. PG-G wrote, edited, and reviewed the manuscript. JK wrote, edited, and reviewed the manuscript. RF reviewed the manuscript. PH edited and reviewed the manuscript. MB edited and reviewed the manuscript. DC edited and reviewed the manuscript. All authors contributed to the article and approved the submitted version.

## Funding

Work for this manuscript was supported by NIH NIAID K08 AI143968 01A1 supported by the Division of Intramural Research, NIAID, NIH.

## Conflict of Interest

The authors declare that the research was conducted in the absence of any commercial or financial relationships that could be construed as a potential conflict of interest.

The handling editor declared a past co-authorship with several of the authors PH, MB.
